# Evaluation of the 2013 Southeast Asian Haze on Solar Generation Performance

**DOI:** 10.1371/journal.pone.0135118

**Published:** 2015-08-14

**Authors:** Mohammadreza Maghami, Hashim Hizam, Chandima Gomes, Shahrooz Hajighorbani, Nima Rezaei

**Affiliations:** 1 Department of Electrical and Electronic Engineering, Universiti Putra Malaysia, Serdang, 43400, Selangor, Malaysia; 2 Centre of Advanced Power and Energy Research (CAPER), Universiti Putra Malaysia, 43400, Selangor, Malaysia; Tsinghua University, CHINA

## Abstract

Pollution in Southeast Asia is a major public energy problem and the cause of energy losses. A significant problem with respect to this type of pollution is that it decreases energy yield. In this study, two types of photovoltaic (PV) solar arrays were used to evaluate the effect of air pollution. The performance of two types of solar arrays were analysed in this research, namely, two units of a 1 kWp tracking flat photovoltaic (TFP) and two units of a 1 kWp fixed flat photovoltaic arrays (FFP). Data analysis was conducted on 2,190 samples at 30 min intervals from 01^st^ June 2013, when both arrays were washed, until 30^th^ June 2013. The performance was evaluated by using environmental data (irradiation, temperature, dust thickness, and air pollution index), power output, and energy yield. Multiple regression models were predicted in view of the environmental data and PV array output. Results showed that the fixed flat system was more affected by air pollution than the tracking flat plate. The contribution of this work is that it considers two types of photovoltaic arrays under the Southeast Asian pollution 2013.

## Introduction

Haze often occurs when dust and smoke particles accumulate in relatively dry air [1–3]. When weather conditions block the dispersal of smoke and other pollutants, they concentrate and form a typically low-hanging shroud that impairs visibility and may become a respiratory health threat. Industrial pollution can result in dense haze, which is known as smog. Since 1991, haze has been an acute problem in Southeast Asia because Indonesian forests are burned to clear land and the resultant smoke is blown by the wind to cover neighbouring countries [4]. Energy losses caused by air pollution on solar panels are widespread, and the situation in each area is different [5, 6]. This study considers the air pollution in Southeast Asia in July 2013. The Southeast Asian haze, which occurred from 13th June to 19th June 2013, affected several parts of the region, including Brunei, Indonesia, Malaysia, Singapore, and southern Thailand [7, 8].

### Influence factor on accumulate dust particles

**Fig 1:**
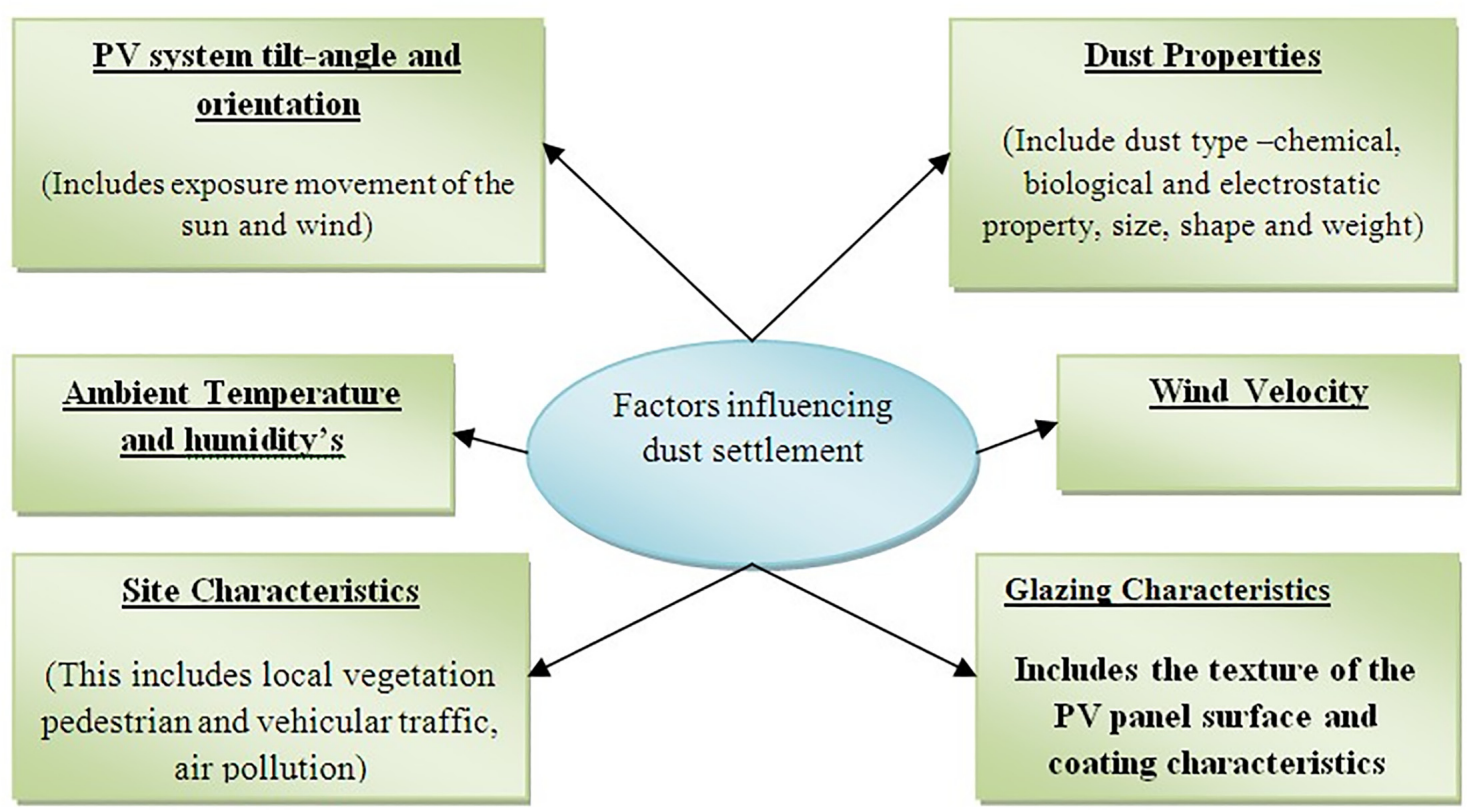
Factors influencing dust settlement. Many parameter influence to deposition of dust on the surface of solar panel. One of the main parameters that influence on accumulation dust is property of dust. Dust characteristics including the type chemical, size shape etc. Wind velocity either can increase or decrease the dust deposition. Local environment is an important part, which need to consider more on site characteristics, ambient temperature and humidity’s. On the other hand, solar panel angle and orientation, glass surface are the parameters which lead to increase the dust on the surface of solar array [9].

The characteristics of soiling accumulation on solar panels are the result of two main parameters that influence each other: the property of dust and the local environment. The local environment includes site characteristics resulting from human activities, built environment, environmental characteristics (type of vegetation) and weather situations [9–13]. The property of dust (chemical, type, weight, shape, and size), is as significant as its accumulation. Similarly, the surface of the solar array plays an important part to accumulate dust on the surface. A sticky glass surface (rough, electrostatic build-up, adhesive residues, furry) is more likely to build up dust than a smoother one. Flat surfaces (horizontal) typically have a tendency to build up more dust than inclined surfaces, but this is depended on the prevailing wind directions. In general, a low-velocity wind encourage dust accumulation on the top of the solar panel while a high-velocity wind, in contrast, disperses dust and has a cleaning effect. However, wind movements can either decrease or increase the accumulation dust on solar panels at particular places of the solar panel [14]. Dust is likely to resolve in areas of low-pressure encouraged by high-speed wind movements over is posed/ perpendicular surfaces. The scattering of dust attributed to the geometry of solar panel and wind movements relies on the characteristics of the dust (type, size, weight, and shape). A framework to recognize the different issues that govern the accumulation of dust on the surface of solar panel is explained (see Fig 1) [9].

Air pollution is one of the factors that directly affect the efficiency of solar generation systems [15]. Numerous studies have focused on the effect of the environment on solar power generation, such as the effect of dust, dust storms, wind speed, angle, and shedding [9]. In 1942, Hottel and Woertz first studied the effect of dust on solar panel performance by investigating the dust accumulation on such a panel [16]. Their three-month test was performed in an industrial area near Boston, Massachusetts that included a power plant and also a four-track railway that was just 92 metres away from the site. They found an average of 1% loss of incident solar radiation caused by dust that accumulated on the surface of the solar panel with a tilt angle of 30°. The maximum degradation reported during the test period was 4.7%. The researchers deduced a correction factor, defined as the ratio of the transmittance from an unclean or exposed glass plate to a clean one, of 0.99, with a 45° tilt angle. This value was adopted and accepted in the design of flat-plate collectors until 1970. In 2001, Soleimani et al [17], studied the effect of air pollution on PV performance. The influence of air pollution is considerable for a large city such as Tehran. The researchers found that the PV power output decreased by more than 60% as a result of air pollution.

**Fig 2:**
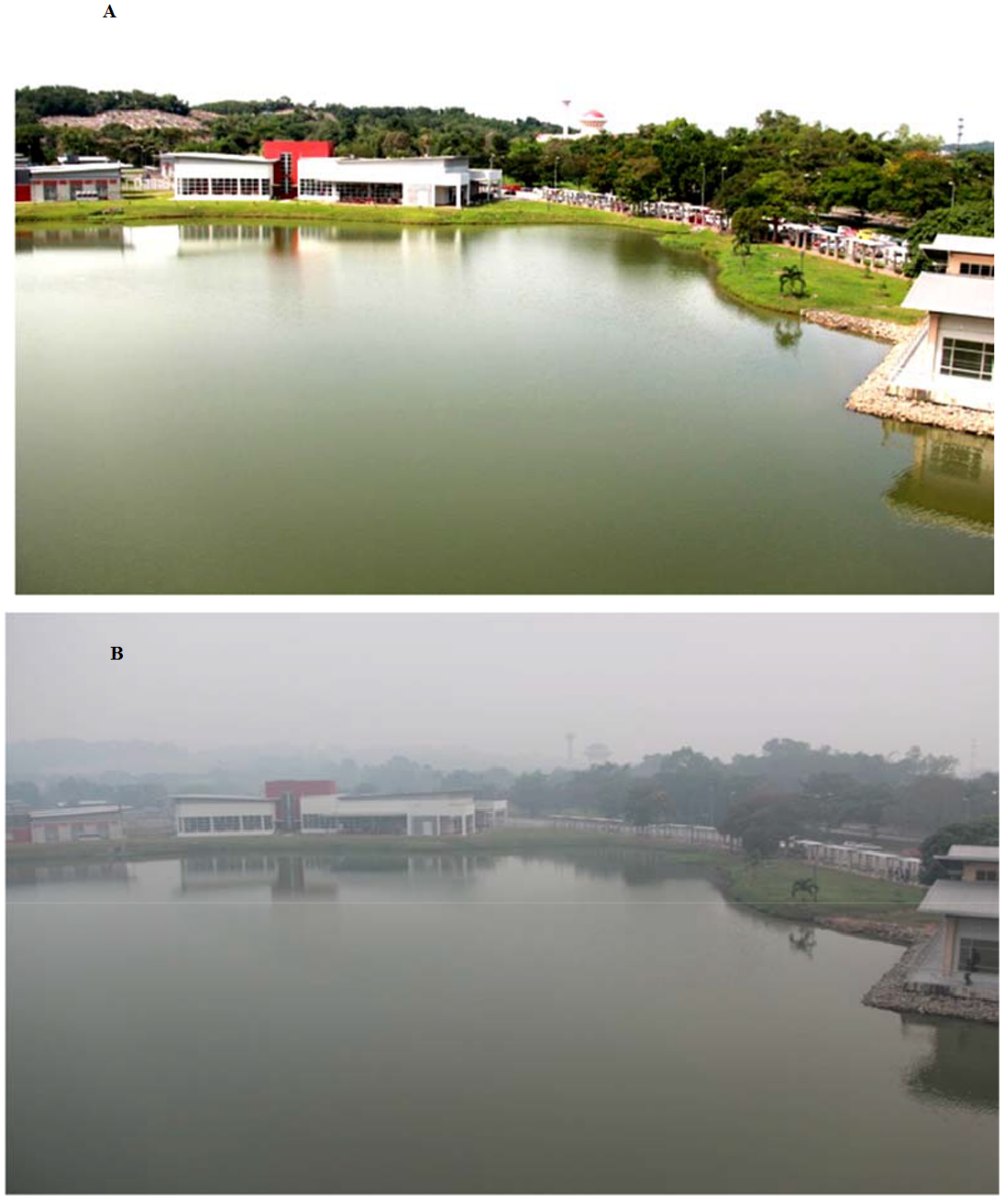
Faculty of engineering (UPM) before haze pollution and during haze pollution. Fig 2A, shows the Faculty of Engineering Universiti Putra Malaysia (UPM) before the haze pollution. This photo was taken by camera on 25^th^ of May 2013, in a day with high resolution just a few days before start haze pollution while the effect of haze pollution in the same place is shown in Fig, 2B, on an unclouded day.

Air pollution can enormously affect PV power generation and cause substantial power dampening during the event. The UPM Faculty of Engineering experienced one such phenomenon during the summer of 2013. Fig 2A shows the Faculty of Engineering Universiti Putra Malaysia (UPM) before the haze pollution while the effect of haze pollution in the same place is shown in Fig 2B on an unclouded day. The effect of this incident will be studied in this paper. Due to the haze pollution a small layer of dust covered the surface of the solar panels. If there is prolonged exposure, the glass surface can be gradually eroded because when the moist dust attaches itself to the surface of the PV glass cover, the major components of the glass begin to experience an acid or alkaline reaction. Over time this can increase the amount of acid or alkaline in the immediate environment so the surface of the glass cover can become bumpy, resulting in a decrease of the PV lifetime.

### Physical principle and research problem

Solar cells are wired in parallel and in series to form a PV Module. The number of series cells indicates the voltage of the solar Panel Module, whereas the number of parallel cells indicates the current. If many cells are connected in series, shading of individual cells can lead to the destruction of the shaded cell or of the lamination material, so the solar Panel Module may blister and burst. In addition, Hajjah et al [18], defined the yield factor (YF) as the annual, monthly, or daily net AC energy output of the system divided by the peak power of the installed PV array under standard test conditions given by the units kWh/kWp. In [19], the yield value is defined as the duration that a PV device would have to operate at its full rated power level in order to generate the same amount of energy that it actually did generate under the actual conditions. It is usually calculated over a day. The capacity factor (CF) is defined as the ratio of the actual annual energy output to the amount of energy that the PV array would generate if it operated at full rated power (Pr) for 24 hours a day for a year.

**Fig 3:**
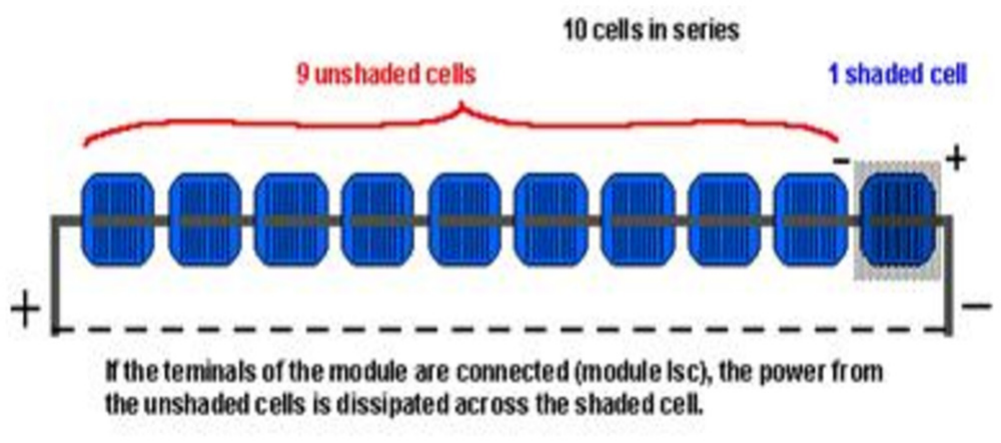
Current flows through shaded cells. Solar panel module is consisting of 10 cells and with one cell shaded and unable to produce any current. As the figure shows, in this condition the shaded cell acts as a resistance to current generated from other cells. This causes the shaded cell to heat up and lead to a hot spot that can eventually damage the module [20, 21].

The physics principle of this research on reduction of energy, some soil patches such as leaves, bird droppings and dirt patches that block some cells of a PV module not the whole, have a severe effect on solar panel modules. Fig 3 shows a solar panel module consisting of 10 cells and with one cell shaded and unable to produce any current. Fig 3 shows in this condition the shaded cell acts as a resistance to current generated from other cells. This causes the shaded cell to heat up and lead to a hot spots that can eventually damage the module [10, 20–23].

This study aims to define the effect of the 2013 Southeast Asian haze pollution on solar generation. This study was conducted from the 1st to the 30th June 2013. The duration of this study was considered sufficient based on the pollution conditions in Malaysia at that time. A practical approach is proposed to compare the power output and energy yield from both arrays. The power output, energy yield, irradiation, temperature, air pollution index and haze particles before and after the haze have been investigated.

## Experimental and Method

To determine the amount of energy losses caused by the Southeast Asian haze on solar panels, the authors used two types of PV array in this study. Both of these were cleaned on the 01st June. The power output of both PV arrays before the haze pollution on the 1st to the 10th June, during the haze period (11th to the 21st) and after the haze pollution till the 30th June 2013, were compared. During this period, the sampling time was every 30 min. SPSS 19.0 for Windows was used to analyse the data. Variables were compared with the descriptive statistics power output for the FFP and TFP arrays. Two-tailed *P* < 0.05 was considered statistically significant.

### Description of photovoltaic arrays

**Fig 4:**
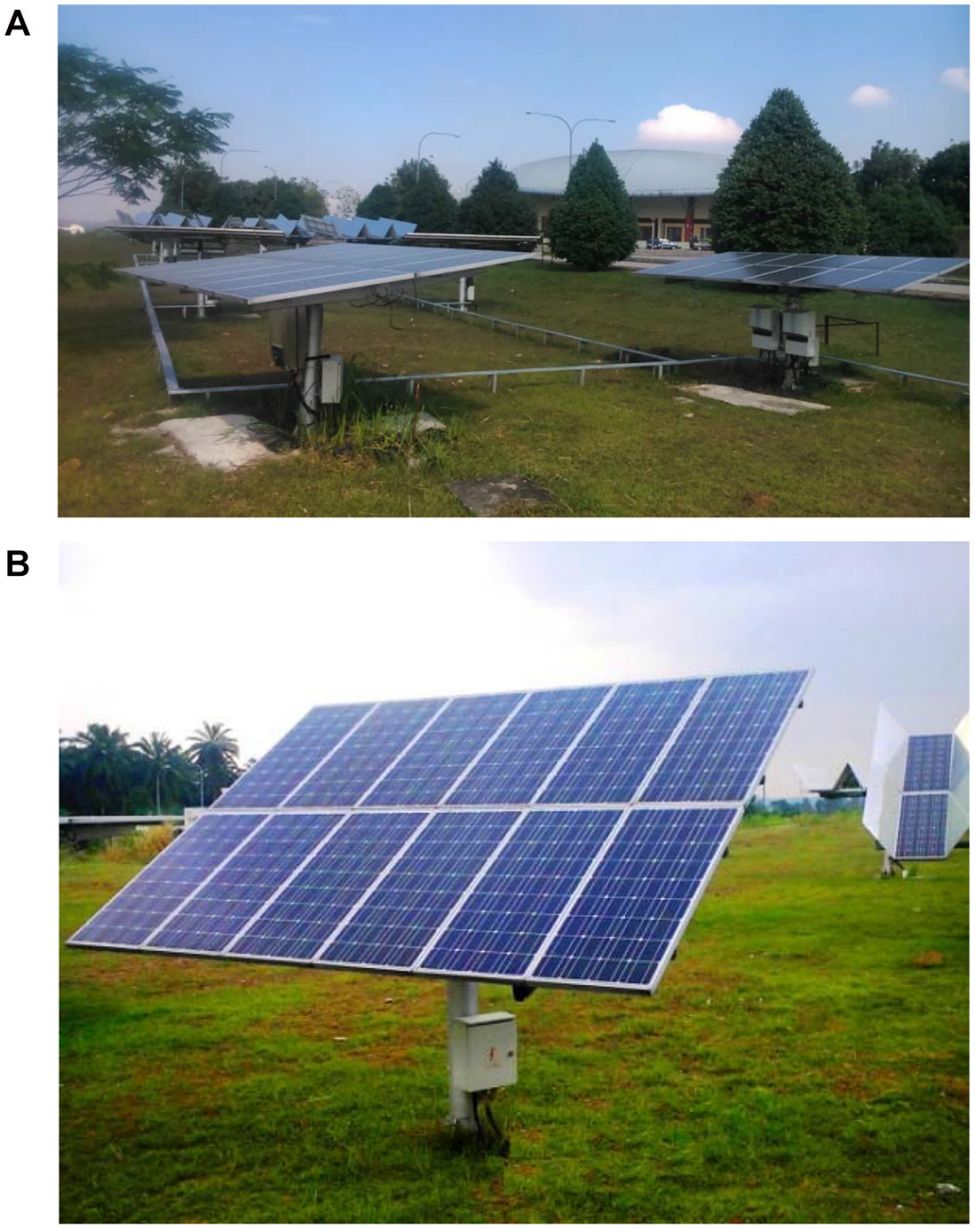
Installed two types of photovoltaic array in UPM. These two of PV arrays consist of two 1 kWp units of a fixed flat photovoltaic (FFP) arrays (Fig 4A). Each array contains 12 modules, which connected series. These PV modules are made of 108 mono crystalline silicon solar cells (9 pcs × 12 pcs) to make a panel 125 mm × 62.5 mm. And another types of photovoltaic is two 1 kWh units of a sun tracking flat photovoltaic (TFP) array which is made up of CEEG 95 watt mono crystalline PV modules (Fig 4B). The tracking flat PV generator system uses series connected configuration known as PV array 12 modules of 95W Mono crystalline PV modules.

The solar arrays are made from mono crystalline silicon CSUN modules for which the data sheet is presented in Table 1. These PV modules are made of 108 mono crystalline silicon solar cells (9 pcs × 12 pcs) to make a panel 125 mm × 62.5 mm. The standard testing condition (STC) is IEE-61215 −1000 W/M, at 25°C. The four solar arrays employed in this study are ground mounted. These two types of PV arrays consist of two 1 kWp units of a fixed flat photovoltaic (FFP) plate (Fig 4A), and two 1 kWh units of a sun tracking flat photovoltaic (TFP) array (Fig 4B). According to Table 1, both types of PV array are made up of CEEG 95 watt mono crystalline PV modules. The PV plates are connected to the distribution grid.

**Table 1:** Characteristics of the PV module.

Appearance		
Characteristics	P_max_	P Nominal
Maximum power(W)	95	92
Voltage (V)	18.3	17.9
Current (I)	5.21	5
Open circuit voltage(V)	22.5	20
Short circuit current(I)	5.56	5.4
Weight (kg)	8	8

Specifications of 1 kW PV arrays shows in this table which made up in CSUN. The maximum power generation is 95, voltage is 18.3 V and current is 5.21 ampere.

**Fig 5:**
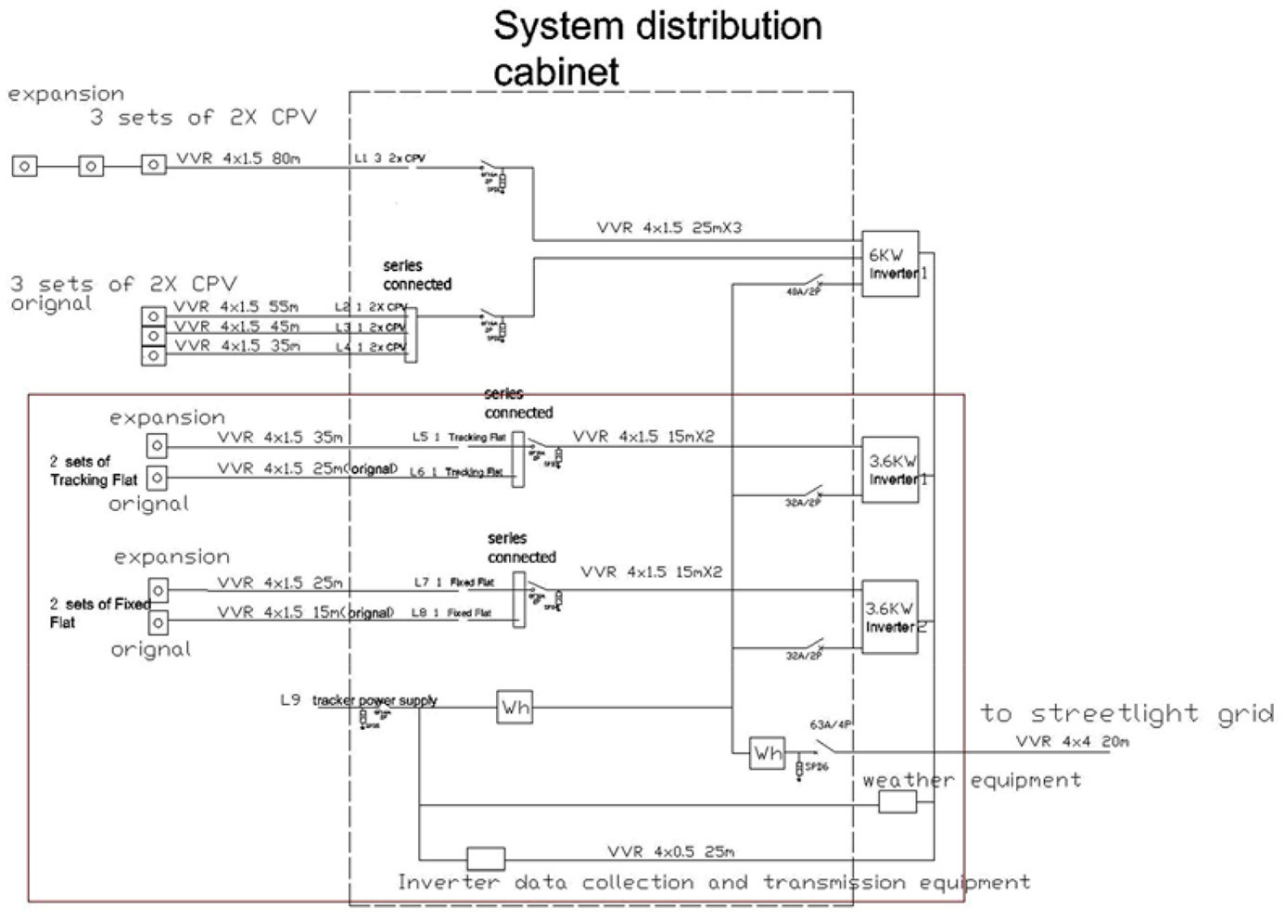
Block diagram of both PV arrays. The diagram of both arrays was shows in read block. The Fixed Flat arrays and Tracking Flat arrays are connected in series to one inverter 3.6KW. The DC side of the two inverters converts the direct current produced by the PV strings into an alternating current that is compatible and synchronised with the grid.

A block diagram of the solar array is given (see Fig 5). Each FFP and TFP arrays is an array consisting of two strings of six modules in series. In addition, both the FFP arrays and TFP arrays are connected in series to one inverter. The DC side of the two inverters converts the direct current produced by the PV strings into an alternating current that is compatible and synchronised with the grid. Finally, the AC side of the two inverters is connected to a double-primary transformer that converts the low voltage output of the inverters (315 V) up to 20 kV corresponding to the nominal voltage of the electrical grid.

In this case, the standard for the performance of both types of solar panel is calculated by referring to the MS-IEC standards and a recent study in [19]. The maximum power output, P_DC_ was recorded for each array by searching for the power value which occurs at 1000 W/m^2^ in the recorded data with a tolerance of approximately 5%. The P_DC_ power is given by multiplying the Voltage DC by the Current DC from the PV module output. In addition, the standard definition of PV array efficiency is used which is the ratio of the output PV array power to the input solar power expressed as a percentage [18, 19]. Hajjah and Khatib [18] considered the energy yield as the daily, monthly, and annual power generation of the arrays.

### Location of installed solar arrays

**Fig 6:**
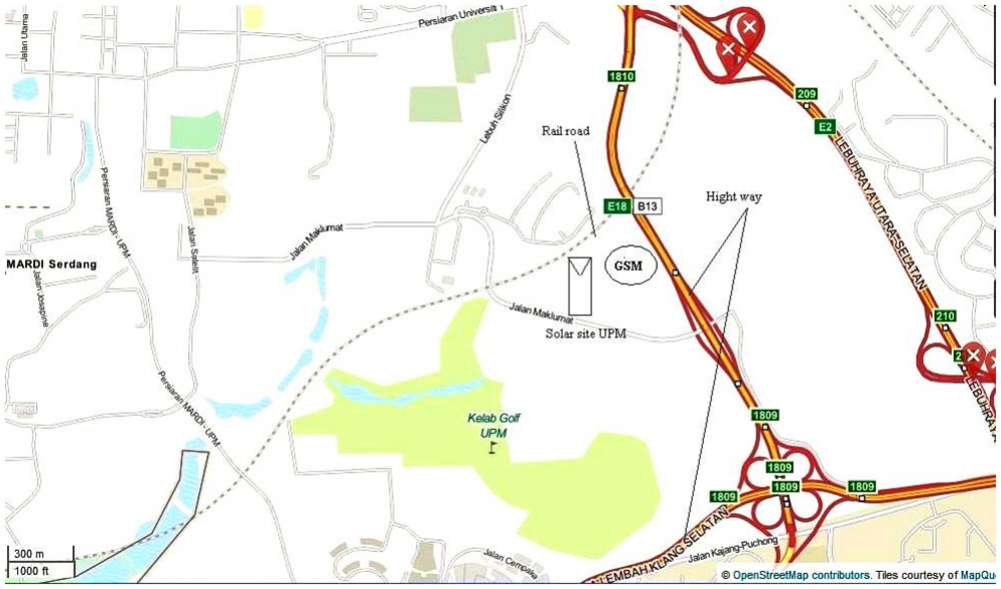
Location of installed both arrays. Solar panels were installed in Serdang, Malaysia, at coordinates 2°59'20"N 101°43'30"E and under tropical-based ground conditions. Two highways with vegetation are located 800 m away, and a new Graduate School of Management building has been constructed near the site.

Four panels of 1 kW-rated PV arrays, namely two FFP and two TFP, were installed in Serdang, Malaysia, at coordinates 2°59'20"N 101°43'30"E and under tropical-based ground conditions. Fig 6 shows the geographical locations of the two PV plates installed at Universiti Putra Malaysia. Each array is south exposed and the tilt angle is 15° (FFT). As shown in Fig 6, two highways with vegetation are located 800 m away, and a new Graduate School of Management building has been constructed near the site [24].

### Data collection and the air pollution index

**Fig 7:**
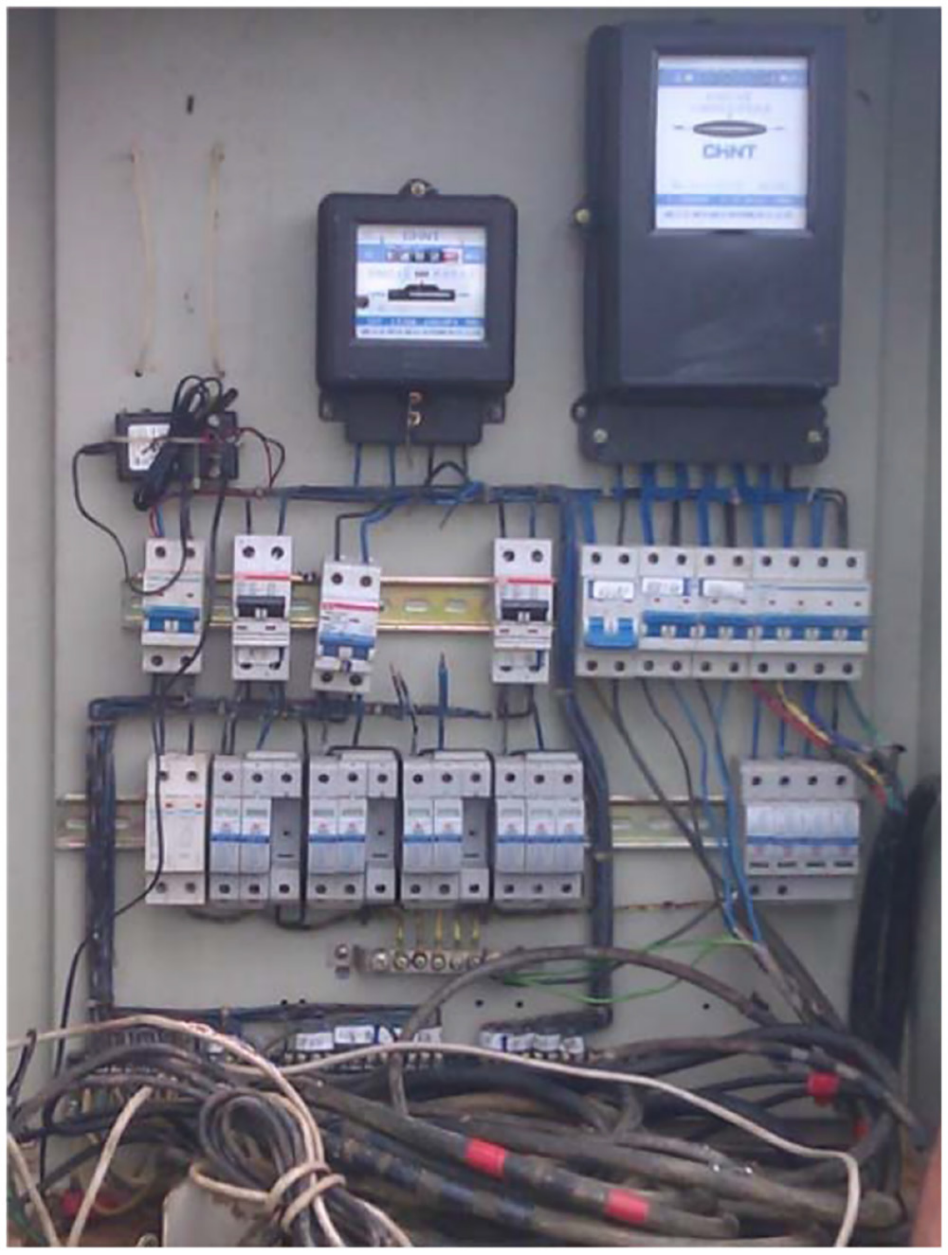
Distribution box (Feeder Pillar). The system is directly connected to UPM electrical distribution line via Feeder Pillar (FP), which links to the Main Switch Board (MSB). The ten units of PV generator are connected to three units of Aurora Inverter system with the capacity of 2 x 3.6 kW and 6.0 kW for the purpose of Grid-tied operation [24].

Two types of data were observed in order to determine the effect of haze on both arrays. The first type of data was collected from the PV arrays such as the voltage (DC), current (DC) and temperature during the PV output (S1 Dataset). The system was directly connected to a UPM electrical distribution line via a Feeder Pillar (FP) (Fig 7) which links to the Main Switch Board (MSB). The data output was connected to a data logger with a GPRS and was monitored online every minute. In this study, data was selected to obtain samples from the first of June to the 30th June 2013(S1 Fig).

**Fig 8:**
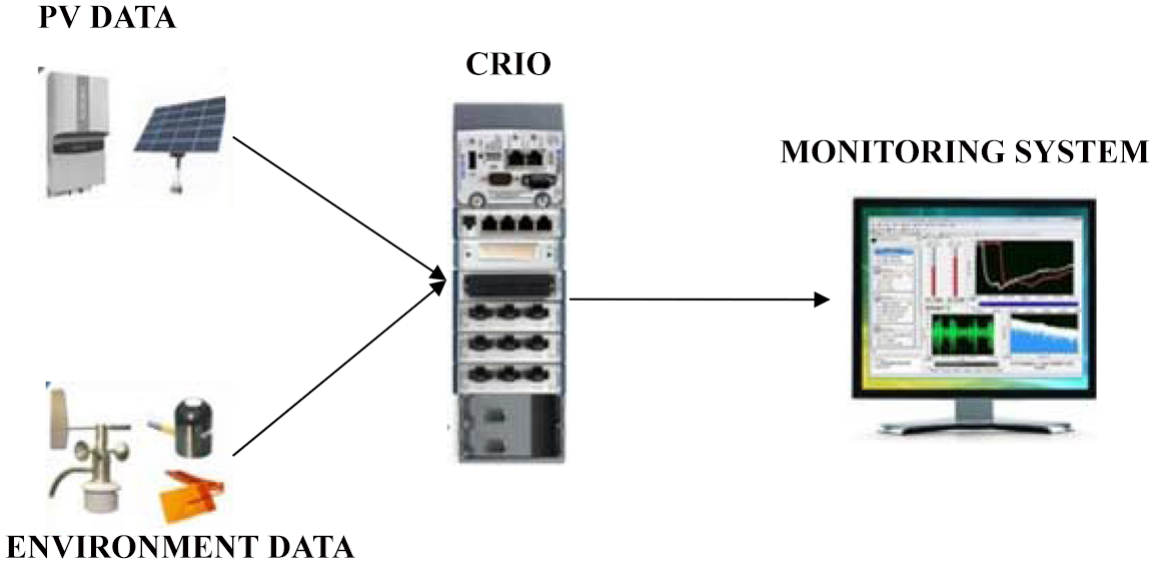
System setup proposed for DART monitoring system. It consists of both online and ground platform interface via Data Acquisition and Real-Time Monitoring (DART) system to capture measurement from multiple input sources and analyse in synchronize mode.

In addition, an automatic weather station data monitoring system has been set up at the site comprising of a wind speed sensor, ambient temperature sensor and solar radiation sensor (S2 Fig). All of these devices and the PV generator outputs were linked directly via a Wireless Network Sensor (WNS) configuration whereby data from the site was transferred to a cloud database through the General Packet Radio Service (GPRS) using 3G cellular communication. The site used the online monitoring system which could be accessed through the www.smartpv.net website. The measurements were taken every minute min in order to consider the uncertain nature of the recorded data. The DART PV monitoring system at the site was designed to capture measurements from multiple sources and analyse visually in real-time and synchronise mode (S1 Fig). The crucial aspect of monitoring rapid fluctuating data flow was technically supported by the Virtual Instrument & System Innovation S/B (VISI) and the process flow is illustrated (Fig 8).

The Air Pollutant Index (API) is an indicator of the air quality status in any particular area. It is calculated based on five major air pollutants which are: **1**- Sulphur Dioxide (SO2), **2**- Nitrogen Dioxide (NO2), **3**- Carbon Monoxide (CO), **4**- particulate matter with a diameter < 10 micron, PM10 and **5**- ground Level Ozone (O3).

The ambient or roadside concentrations for each pollutant, over different averaging times, are converted into an index value. The most popular approach is often called the US-based system. Pollutant concentrations for each pollutant are transformed onto a normalised numerical scale of 0 to 500, with an index value of 100 corresponding to the primary National Ambient Air Quality Standard (NAAQS) for each pollutant [25]. Countries such as Taiwan, Singapore, Thailand, Malaysia, China, Hong Kong, and South Korea have designed their API systems based on the US model. The key reference point of these systems is the index value of 100, which is based on the short-term air quality standards of the respective jurisdictions. Very often, an index value of 50 is anchored to the long-term air quality standards.

### Air pollution Index and haze particles

The haze materials in June 2013 included ash and soot, of which major haze components are particulate matters CO, SO_2_, O, Fe, Ca, NO_3_, and Al. The size of these particles was less than 12 Microns. Table 2 shows the air pollution index during June 2013. The Air Pollution Index (API) which is used in Malaysia is a simple and common way to describe the air quality. It is computed from various sets of air quality data [26]. The Malaysian government classifies air pollution levels at different levels. If the API is under 50, a state of ‘good’ is declared in the reporting area and it means that there is low pollution without any bad effect. If the range of API is between 50 to 100 it is named as ‘moderate’. Moderate refers to pollution that does not pose any bad effect on health and the environment. When the API becomes 100 to 200 it is called ‘unhealthy’, as it can worsen the health condition of a person breathing it, and finally if the API exceeds 500, a state of ‘emergency’ is declared in the reporting area. Usually, this means that non-essential government services are suspended, and all ports in the affected area are closed. However, according to the dates (Table 2), most portions of the period of study were classified as ‘unhealthy’ which is highlighted with Italic in the second column. However at some other times in each day, there were less unhealthy periods and also at the end of the haze problem, which took place over three days, there were some extremely unhealthy (hazardous) days or ‘emergency’, which is highlighted in bold (Table 2).

**Table 2:** API readings during the hazardous period.

Date	Max	Min	Mean
**11**	100	42	75
**12**	*105*	49	73
**13**	*110*	51	84
**14**	*107*	48	96
**15**	*132*	45	101
**16**	*151*	51	105
**17**	*183*	55	111
**18**	*189*	61	114
**19**	*197*	59	125
**20**	***211***	62	135
**21**	***231***	65	143

**Good (0–50); Moderate (51–100); Unhealthy (101–200); hazardous (200–300).**

### Characterisation of haze particles

The particles that cause the haze pollution can originate from many sources, some of which are natural and some anthropogenic. Natural sources include the oceans, forests and the surface of the ground. However, the majority of the particulates are from human activities, which include open burning, land clearing, vehicular use and combustion of fossil fuels in industrial boilers. In this current case under consideration, most of the particles came from forest fires in Indonesia. Scanning electron Microscopy (SEM), Atomic Force Microscope (AFM) and Energy-dispersive X-ray spectroscopy (EDX) were applied in this study in order to consider the type and size of the haze materials.

The dust particles were analysed twice in the month, by sending the dusty PV module to the laboratory. The thickness of the dust particles collected on the module was calculated using the following basic formula:

Thickness = Volume of dust / Area of solar array

where the volume of dust collected was equal to the dust collected divided by the density of the dust. The area of the solar array in this case was 3.6m × 2.4m (S3 Fig) for all the arrays.

### Data analysis

This study was conducted from 1^st^ to 30^th^ of June 2013. The duration of this study is considered sufficient based on the pollution conditions in Malaysia. A practical approach is proposed to compare the power output from both arrays. The power output before and after the haze is compared. Power output were calculated by multiple current and voltage (DC) which collected by online monitoring system (S1 Fig). The mean power output was calculated and used in statistical analyses for both arrays. PV characteristics data and environment data were combining, because to consider the both arrays under same weather condition. Power output, irradiation, temperature of the arrays and thickness of dust, were selected per each date. Using the SPSS version 19 which bought by university Putra Malaysia to analysis the power output, to find correlation between variable by using scatter plot and finding the best fit model to predict power output despite haze pollution by employed multiple regression models [27].

### Model prediction and validation

To evaluate the effect of all environmental factors on the power output of the FFP and TFP arrays, multiple regression analysis was applied. Prior to the analysis, the whole amount of data was categorised randomly into sets including 85% and 15% for modelling and validation respectively. The results of this analysis can be used to develop a model for prediction of the power output for both arrays.

## Results and Discussion

### Output Power

**Fig 9:**
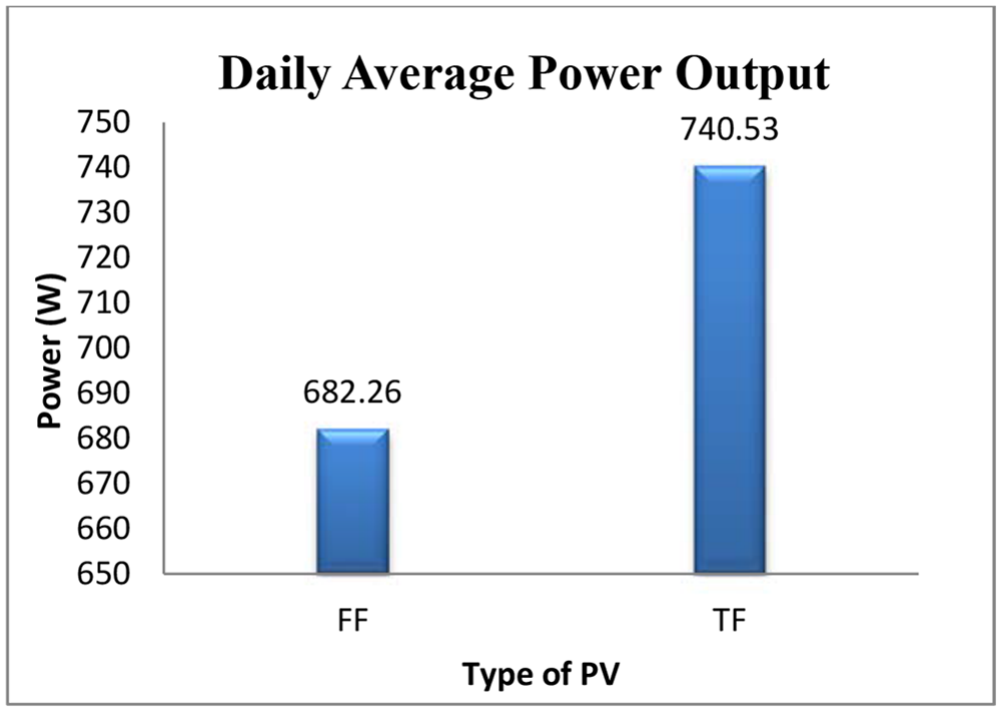
Daily average generations for the both proposed system. The daily average generation for fixed flat PV and tracking flat arrays during June 2013 compared. Tracking flat with average 740.53 W has more generation rather than fixed flat array with generation around 682.26 W. it is seen that during the 30 days the average generation of FFP arrays was around 58 W less than the TFP arrays.

Output power were calculated by multiple current and voltage which collected by monitoring system. The mean output power was calculated and used in statistical analyses for both arrays. The nominal power generation for each PV array (1 kW) is estimated to be 1 kW based on module performance under the STC. Table 3 shows the maximum, minimum, and mean power generated for each array during the haze pollution. It shows that the maximum and minimum generations are 1109 W and 419 W for the TFP and 933 W and 292 W for the FFP arrays respectively. According to the fourth column in Table 3, it is seen that during the 30 days the average generation of FFP arrays was around 58 W less than the TFP arrays (see Fig 9).

**Table 3:** Statistical generations of both arrays.

Plate	Min(W)	Max(W)	Mean(W)	STD
(FFP)	292	933	682.26	178
(TFP)	419	1109	740.53	195

Minimum (Min), maximum (Max), mean and standard deviation (STD) during the haze pollution for the both PV arrays recorded. It is shows that, TFP arrays have highest generation rather than FFP with average generation 740 W.

Table 4 illustrates the average, minimum and maximum daily generation, before, after and during the haze pollution for the FFP. The daily average generation during the haze pollution was lower than before and after the haze period by 216 W and 359 W respectively.

**Table 4:** Descriptive Statistics output power for the FFP arrays.

	Min(W)	Max(W)	Mean(W)	S.D
Before H	629.73	845.41	714.45	18.1
Haze P	292.31	633.10	498.75	38.7
After H	767.24	933.98	857.55	11.0

Minimum (Min), maximum (Max), mean and standard deviation (STD) before, after and during the haze pollution for the both FFP arrays recorded. It is shows that, the average output power reduced from 714.45 W before the haze pollution to 498.75 W during the haze, by the end of haze pollution since the rain coming at 21st of June the average generation going up to 857.55 W. ***Before haze***: starting from 1st to 10th June; ***Haze period***: starting from 11th to 21st June; ***After haze***: starting from 22nd to 30th June.

For the TFP arrays (see Table 5) it can be seen that the average daily generation during the haze pollution is lower than before and after the haze period by 189 W and 387 W respectively. In overly it can be stated that the amount of loss caused by the haze pollution in the TFP arrays is lower than for the FFP arrays. This is due the horizontal surface that trend to accumulated dust on the surface of Fixed Flat arrays, the result of this study similar with previous research such as that by Nashar [28].

**Table 5:** Output power for the TFP arrays.

	Min(W)	Max(W)	Mean(W)	S.D
Before	622.18	922.94	744.53	17.21
Haze	419.93	721.08	555.74	37.25
After	731.31	1109.15	942.29	6.05

Minimum (Min), maximum (Max), mean and standard deviation (STD) before, after and during the haze pollution for the both TFP arrays recorded. It shows that, the average output power reduced from 744.53 W before the haze pollution to 555.74 W during the haze, by the end of haze pollution when the rain coming at 21st of June and washed the surface of arrays, the average generation was going up to 942.29 W. As compared output power from TFP arrays with FFP arrays it was a show that, the TFP arrays has better performance. ***Before haze***: starting from 1st to 10th June; ***Haze period***: starting from 11th to 21st June; ***After haze***: starting from 22nd to 30th June.

**Fig 10:**
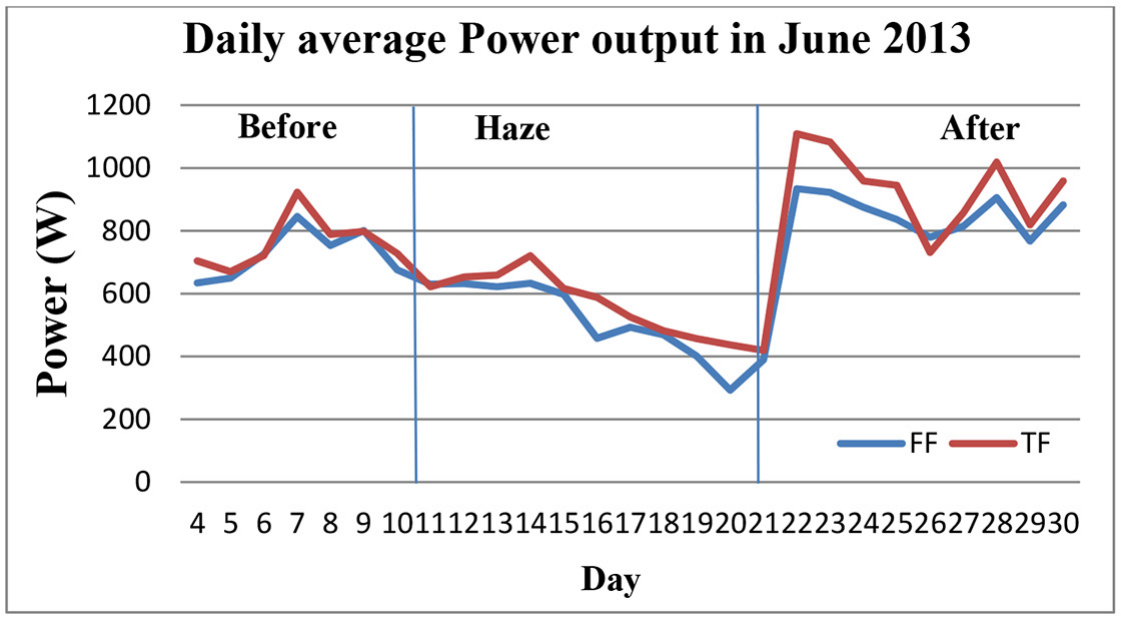
Output power in June 2013. Two types of PV array are compared during June 2013, which both arrays face with south Asia air pollution. In order to investigate effect of haze pollution on output, power generation during the haze compared with before and after the haze pollution. The figure shows that, before haze pollution both arrays have normal performance but on 20^th^ June the haze decreased the output power to 292 W. However, on 21^st^ June 2013, it suddenly increased to 1109 W because the surfaces were washed by rain.

**Fig 11:**
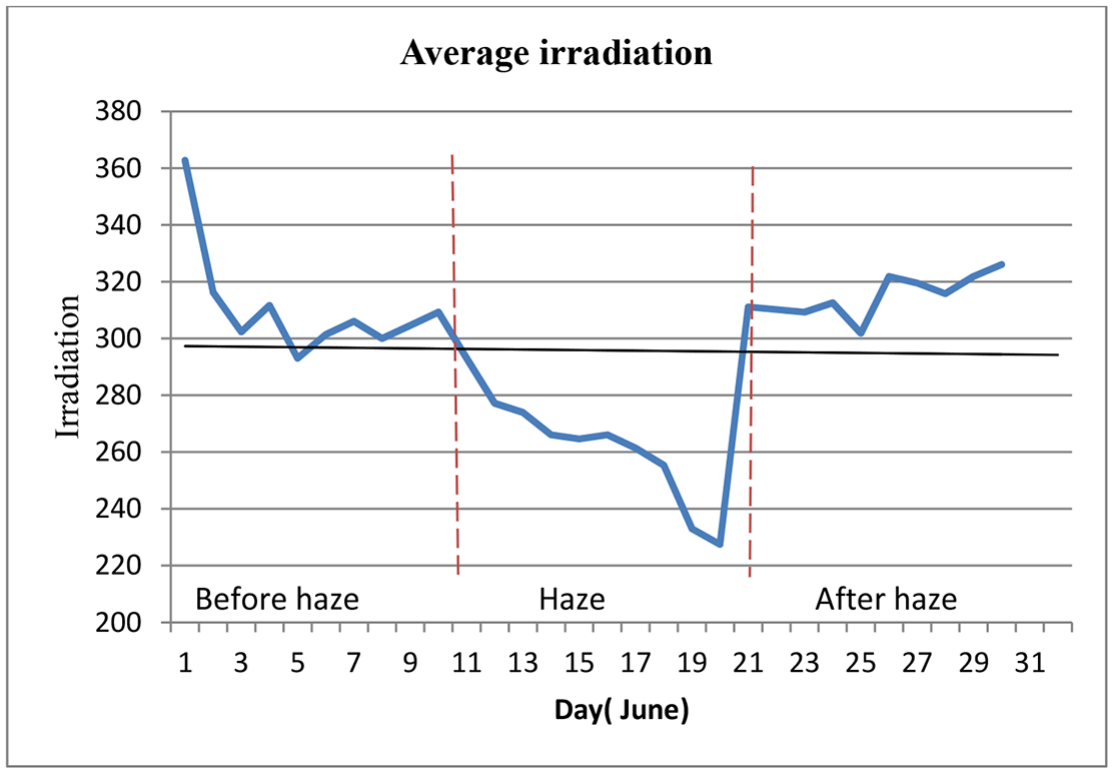
Solar irradiation in June 2013. irradiation suddenly reduces from 11th June, which is when the haze pollution started, till the rain came and cleaned both arrays on 21^ST^ June 2013.

**Fig 12:**
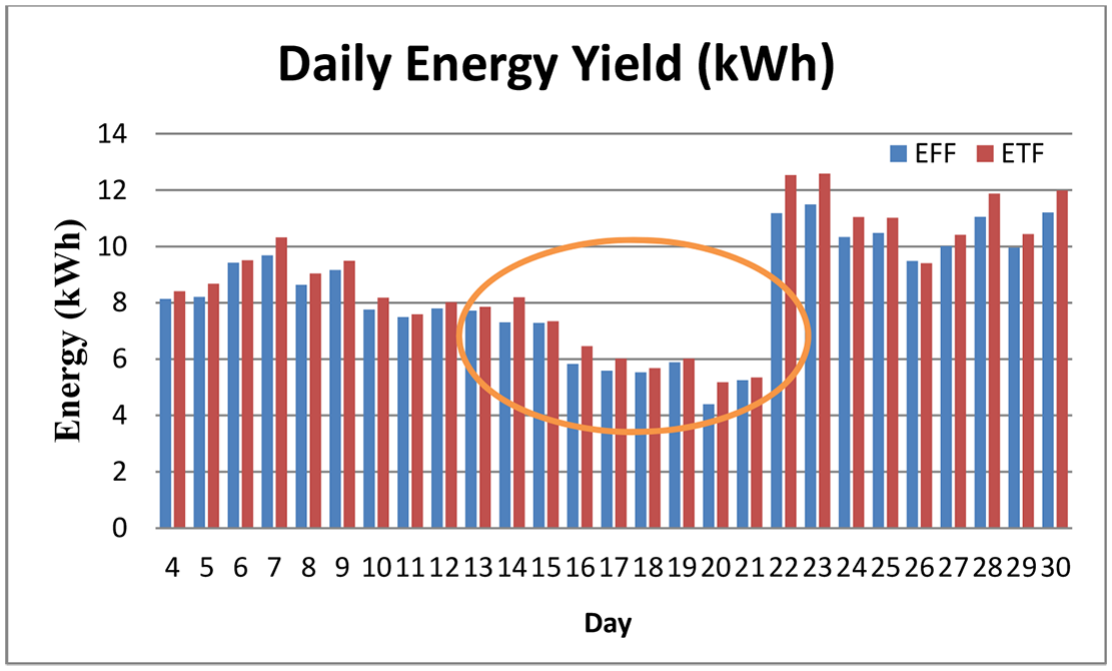
Energy output in June 2013. total energy yield for FP and TF arrays were 238 and 226kWh respectively. Energy yield in June 2013 also have similar performance as output power.

Fig 10 illustrates the results of both the PV array output power within the period that the surfaces were polluted by haze. Initially, on 20th June the haze decreased the power output to 292 W. However, on 21st June 2013, it suddenly increased to 1109 W because the surfaces were washed by rain. This result shows that the PV array can perform sufficiently after the haze and a slight rain is enough to clean the surfaces and again optimise the system efficiency. In addition, when the two types of solar arrays generation were compared, the TFP array with an average daily generation of 740 W performed better than the FFP arrays. Fig 11 shows the average solar irradiation in June 2013. The average irradiation from the sun decreased to 225. With an increase in the pollution in the air the irradiation reduced to a low level, which consequently decreased the output power and efficiency of both arrays. The energy yield also followed the same trend as the power output and showed a similar behaviour. In Fig 12 the total energy yield for the FFP and TFP arrays was 238.01 kWh and 226.30 kWh, respectively. The daily average energy before the haze was 8.59 kWh per day, during the haze it was 6.5 kWh and after the rain which arrived on 21st June the daily average energy yield was 10.57 kWh.

**Fig 13:**
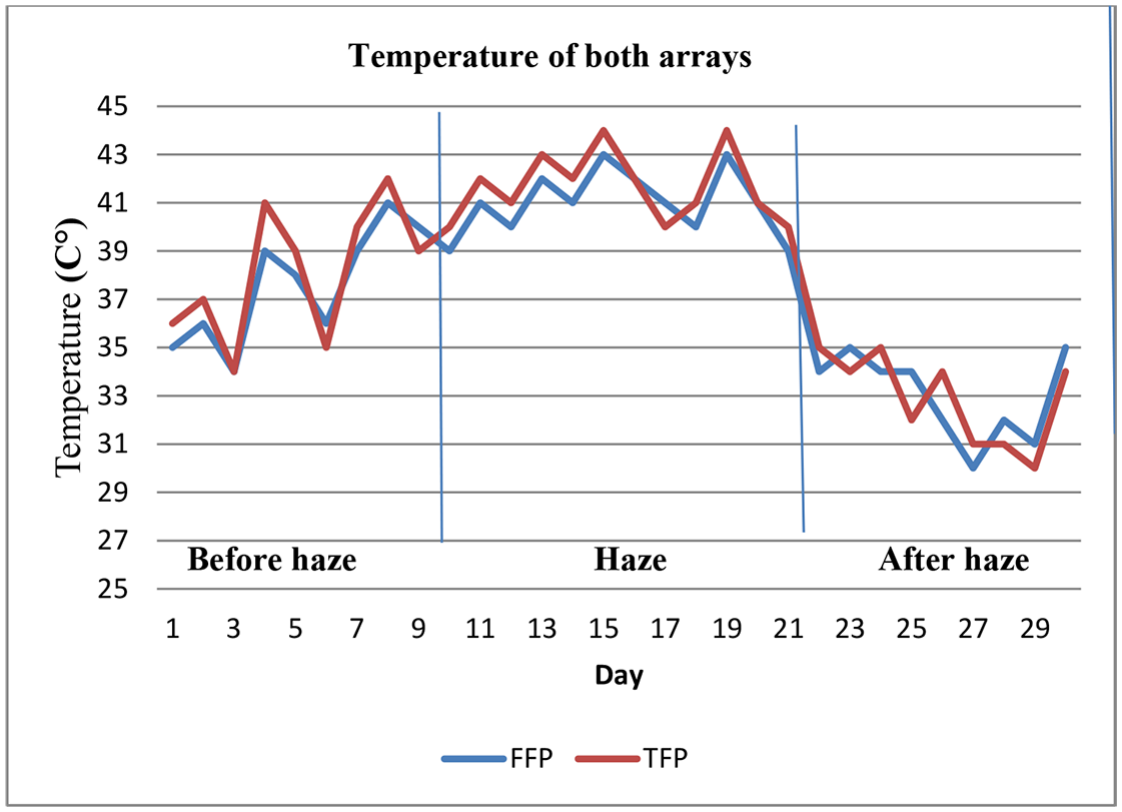
Daily average temperature for both PV arrays was recorded. This figure shows average temperature both arrays during haze pollution. By increase haze pollution temperature of arrays also increased.

Fig 13 shows the average temperature of both arrays during the haze pollution. The average temperature for the FFP arrays was slightly higher than that for the TFP arrays. After the increase in haze pollution from 11^th^ June, the temperature of both arrays also increased. This was caused by accumulated dust material from the haze pollution on the surface of both arrays. According to the Table 6, FFP arrays has highest temperature rather than TFP arrays on the other hand the lowest temperature recorded from TFP arrays with 20.64°C. The result shows that, the average temperature in FFP arrays highest than TFP arrays with 41.29 and 39.93°C, respectively.

**Table 6:** Temperature measurement for both arrays during haze pollution.

	Min(°C)	Max(°C)	Mean(°C)	S.D
T _**TFP**_	20.64	56.12	39.93	3.91
T _**FFP**_	21.00	58.34	41.29	3.43

Temperature of both arrays during the June 2013 was compared in this table. Maximum, minimum, mean and standard deviation of both arrays was measured. The result shows that, the average temperature in FFP arrays highest than TFP arrays. This is can be one of the reasons lead to reduce output power.

### Correlation between variable

Scatter plots can be used to display the relationships between variables. To look at the relationship between two variables, a bivariate scatter plot can be created for each pair of variables. In this part, the relationship between the output powers from both arrays is considered with respect to the environmental data. The best fit for the power output (TFP array) was found to be:

$$Y=2.0001\text{ X +171.54 }R^{2}=0.7757$$ (1)

Where Y is the output power of the TFP array and x is the irradiation from the environment. There was a strong, positive correlation between the power output and the irradiation (IRD). Increases in irradiation were correlated with increases in the rating of the output power from the TFP array. The best fit for the FFP array was found to be:

$$Y=1.7333\text{ X +206.33 }R^{2}=0.7466$$ (2)

**Fig 14:**
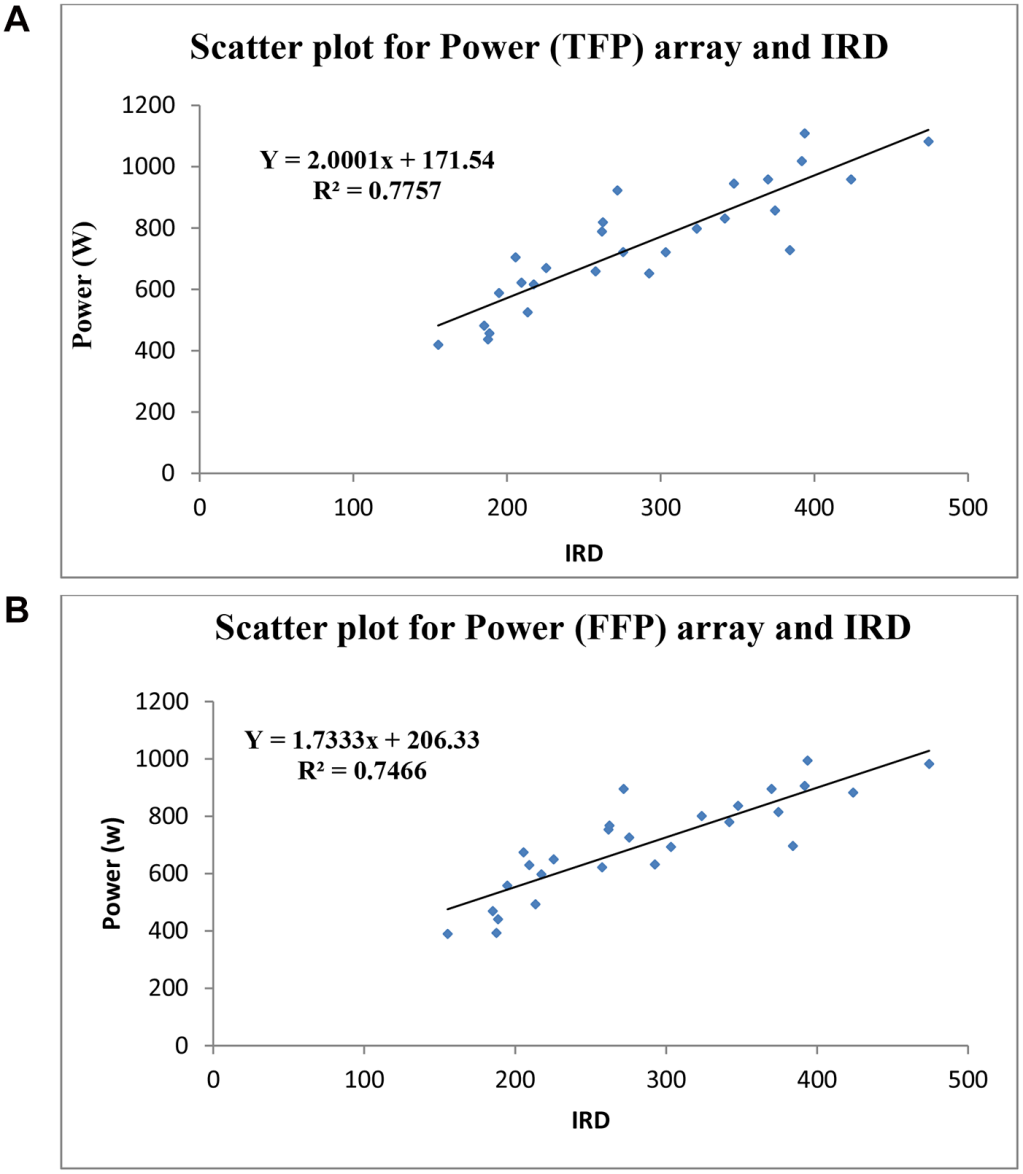
Relation between output power and irradiation. For TFP arrays, there was a strong, positive correlation between the output power and the irradiation (Fig 14A). Increases in irradiation were correlated with increases in the rating of the output power from the TFP arrays (R2 = 0.77). However; Fig 14B shows correlation for FFP arrays, there was a strong, positive correlation between the power output and the irradiation (R2 = 0.74).

This result indicates that the TFP arrays have a stronger relation with the output power (See Fig 14A and 14B).

## Multiple Regressions

The results of the above analysis can be used to develop a model for the prediction of the output power for both arrays. Data for the duration of 01st April until 30th November 2013 was available including the following characteristics: IRD (irradiation), T_e_ (temperature of environment), T_a_ (temperature of array) and humidity of the environment and dust thickness.

The final model contains all predictors including humidity, irradiation, temperature array thickness and temperature environment. Table 7 shows the model summaries for both arrays. The R^2^ of the model is a measure of how much the variability in the output power is accounted for by the predictors. For this model the R^2^ value was 0.895 and 0.901 for the TFP and FFP arrays respectively, which means that the predictors accounted 89.5% and 90.1% of the variation in the output power for both arrays. The adjusted R squared also gives an accurate idea of how well the proposed model can be generalised. Regression coefficients were found by following two regression models:

$$FFP=62.461+T_{FP}\times 11.52+IRD\times 0.861+R\times 68.99-DT\times 19667.532$$ (3)

$$TFP=21.709+T_{TP}\times 11.17+IRD\times 1.104+R\times 91.34-DT\times 17288.051$$ (4)

where is T_FP_ and T_TF_ are the temperature of both the arrays, IRD is the irradiation, R is the rain, and DT is the dust thickness which accumulate on the surface.

**Table 7:** Model summary for both arrays.

**Array**	**R**	**R Square**	**Adjusted R** ^**2**^	**Std. Error**
TFP	0.949	0.901	0.883	59.21
FFP	0.946	0.895	0.876	68.32

The R^2^ of model, which is a measure of how much, is the variability in the output power is accounted for by predictors, and for this model its value is 0.949 and 0.946 for TFP and FFP arrays, respectively. Adjusted R square also gives an accurate idea of how well our model can be generalized.

**Fig 15:**
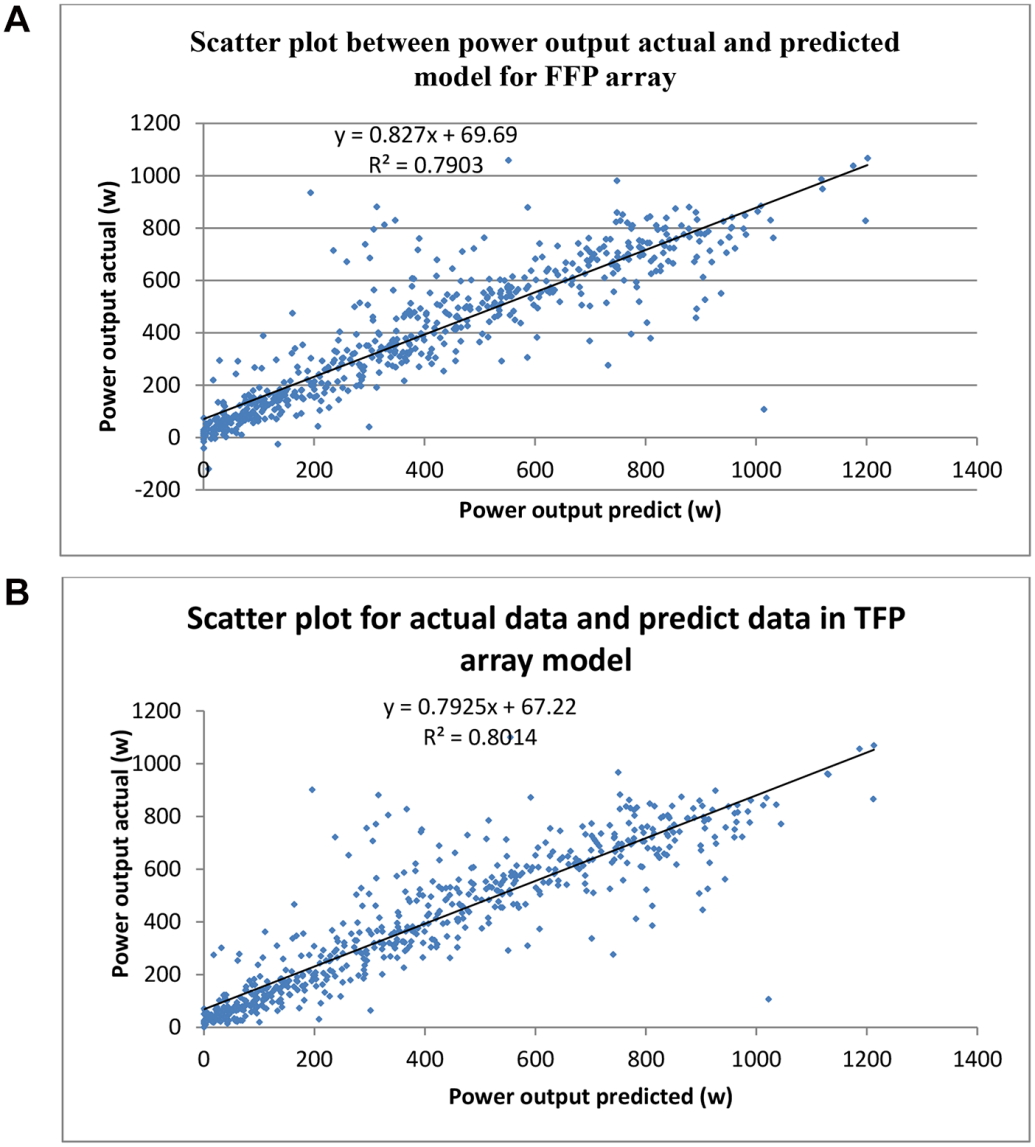
Scatter plot for actual data and predicted data. In the FFP array a scatter plot was chosen to show the model prediction fits with the predicted data (R^2^ = 0.7903). This is show that model prediction for FFP arrays has strong predicted value of the target (Fig 15A). Also scatter plot was chosen for TFP arrays, to show the model prediction fits with the predicted data (R^2^ = 0.8014). This is show that model prediction for TFP arrays has strong predicted value of the target.

In order to validate the model, the validation data set (15% of data selected at random from 01st April until 30th November 2013) was applied. The scoring functions are the types of “scores” available for the selected model. For example, the predicted value of the target, the probability of the predicted value, or the probability of a selected target value. A scoring wizard was employed to obtain a predicted value for both sets of data (the actual data and the predicted data). A scatter plot was chosen to show the model prediction fits with the predicted data. The scatter plot between the output power actual data and the predicted data with R^2^ = 0.7943 for the FFP arrays (Fig 15A). Similarly, a scatter plot for the actual data and the predicted data for the TFP array (Fig 15B).

## Conclusion

In this study, the effect of the 2013 haze in Southeast Asia on the energy yield of TFP arrays and FFP arrays has been investigated. Consequently, the operations of both the TFP and FFP arrays installed at Universiti Putra Malaysia before, during and after the haze were monitored to determine the behaviour of both systems. The results indicated that the effect on PV generation was strongly dependent on the haze pollution. The losses caused by pollution during the 30 days, the average generation of FFP arrays was around 58 W less than the TFP arrays. To obtain this result, more than one dataset per month was analysed and the different environmental elements were applied to the same PV plates. In order to predict the model, multiple regressions was employed despite the dust thickness and environmental data. According to the experimental data, the tracking flat plate (TFP) is the most suitable plate for tropical areas.

## Supporting Information

S1 Dataset Data availability. Data in this file include data from PV arrays and data from weather station. Data from PV arrays include voltage, current and temperature. Each day, data collected from 7 Am until 7 Pm interval each mint for both FFP and TFP arrays. In this file including 27 data set from PV arrays per each day. Another type of data collected from weather station which is including irradiation, temperature, humidity, etc. Data was collected from weather station 24 hour interval each 30 mint. Using the SPSS version 19 which bought by university Putra Malaysia to analysis the power output, to find correlation between variable by using scatter plot and finding the best fit model to predict power output despite haze pollution by employed multiple regression models.
(RAR)

S1 Fig Monitoring system for collecting PV arrays data. In order to get data from the PV array, three sensors of voltage, current and temperature are used, which send data one-minute intervals to the server. The sensors sense the voltage, current and temperature of two clean and dusty PV arrays, and send them to data logger and sends these data to website via GPRS so that user can check the power station running status by logging to website www.smart-pv.net. The data are downloaded from the data logger at one-minute intervals. The DART PV monitoring system at the site is designed to capture measurement from multiple sources and analyze visually in real-time and synchronized mode. The crucial aspect of monitoring rapid fluctuating data flow is technically supported by Virtual Instrument & System Innovation S/B (VISI) and the process flow is illustrated in Figure.
(TIF)

S2 Fig Environment weather station. The environment has a significant impact on accumulation of dust on PV array. In order to get the effect of environment on the solar array we need to collect metrological data (Meteorological Department of Malaysia) such as temperature, irradiation, wind speed, humidity, and air pollution of PV site in UPM. As mentioned before, the sensors and data logger are used to collect these data, at intervals of one minute. The weather station was installed at two-meter height from the ground also the sample data were collected from 31^st^ July to 1^st^ August 2013. Data were collected over 24 hours but for analysis, data selected were for the period from 7 am to 7pm (during the sunlight hours).
(TIF)

S3 Fig Drawing and Dimensions of PV module. (TIF)
