# Supplementary figures and images for: Evaluation of the 2013 Southeast Asian Haze on Solar Generation Performance
Source: PLoS One. 2015 Aug 14;10(8):e0135118. doi: 10.1371/journal.pone.0135118 (PMC4537281; doi:10.1371/journal.pone.0135118)

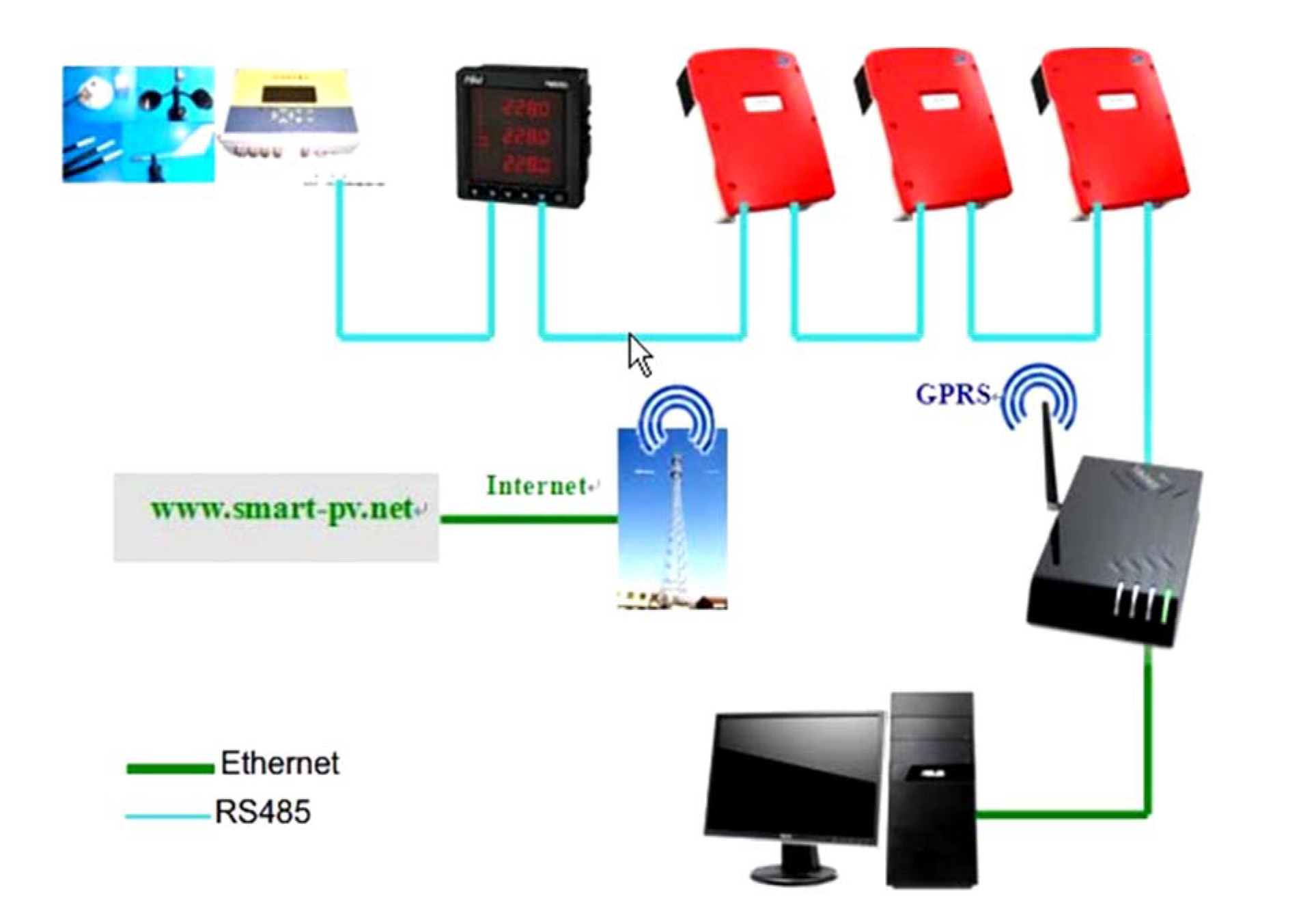

Supplement: S1 Fig — In order to get data from the PV array, three sensors of voltage, current and temperature are used, which send data one-minute intervals to the server. The sensors sense the voltage, current and temperature of two clean and dusty PV arrays, and send them to data logger and sends these data to website via GPRS so that user can check the power station running status by logging to website www.smart-pv.net. The data are downloaded from the data logger at one-minute intervals. The DART PV monitoring system at the site is designed to capture measurement from multiple sources and analyze visually in real-time and synchronized mode. The crucial aspect of monitoring rapid fluctuating data flow is technically supported by Virtual Instrument & System Innovation S/B (VISI) and the process flow is illustrated in Figure. (TIF) [file pone.0135118.s002.tif]

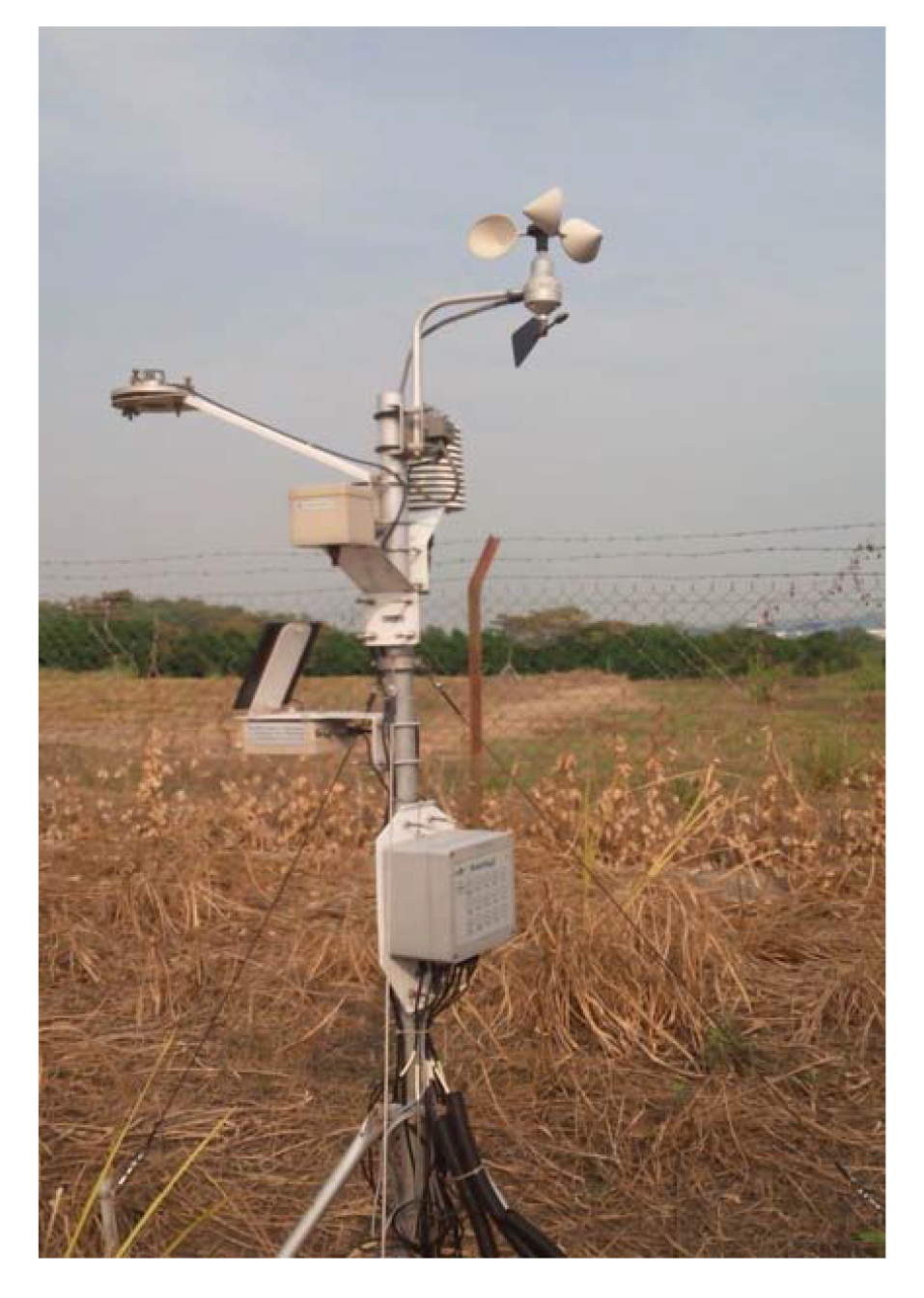

Supplement: S2 Fig — The environment has a significant impact on accumulation of dust on PV array. In order to get the effect of environment on the solar array we need to collect metrological data (Meteorological Department of Malaysia) such as temperature, irradiation, wind speed, humidity, and air pollution of PV site in UPM. As mentioned before, the sensors and data logger are used to collect these data, at intervals of one minute. The weather station was installed at two-meter height from the ground also the sample data were collected from 31st July to 1st August 2013. Data were collected over 24 hours but for analysis, data selected were for the period from 7 am to 7pm (during the sunlight hours). (TIF) [file pone.0135118.s003.tif]

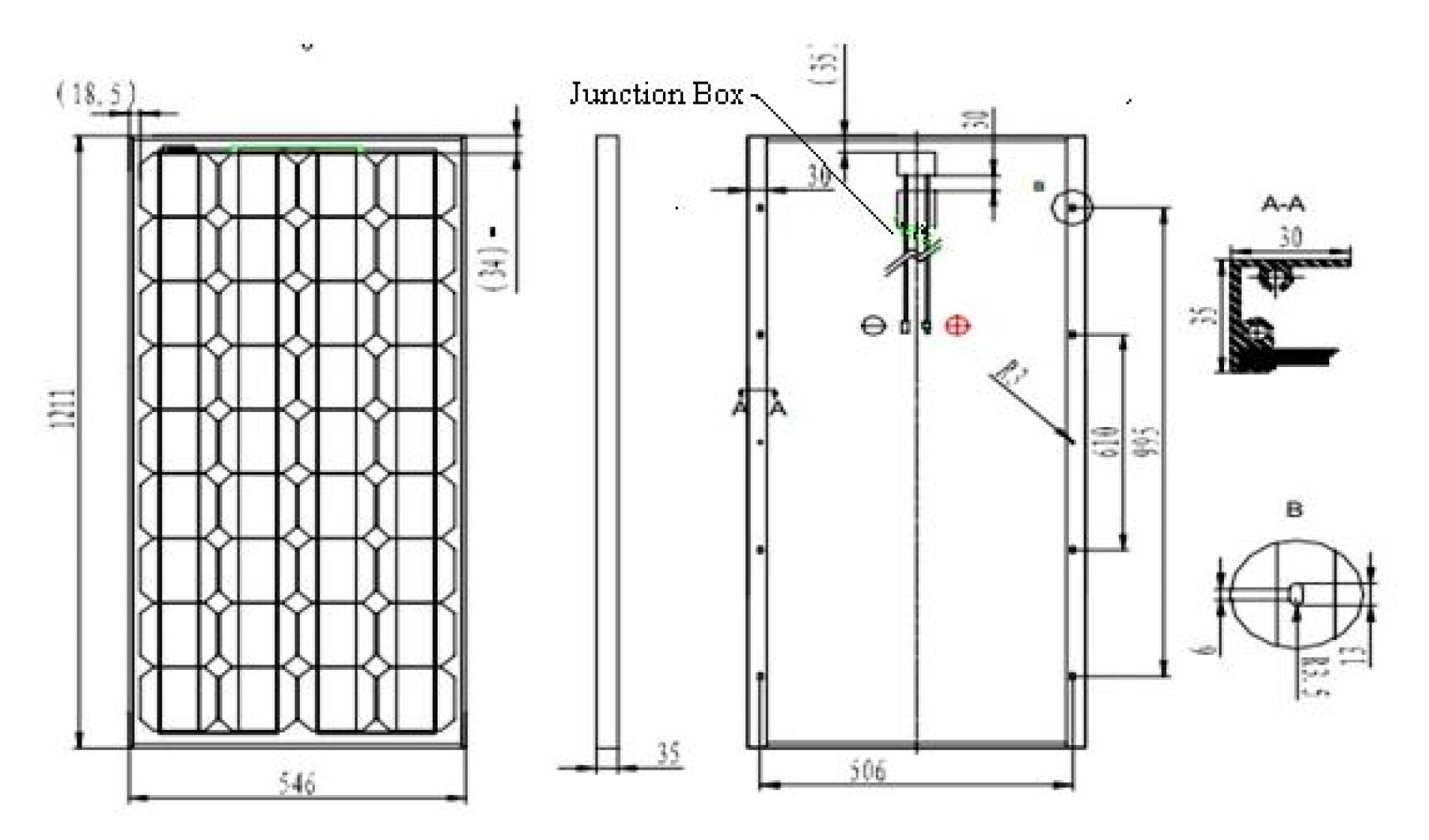

Supplement: S3 Fig — (TIF) [file pone.0135118.s004.tif]
